# The Acute Effects of Single or Repeated Bouts of Vigorous-Intensity Exercise on Insulin and Glucose Metabolism during Postprandial Sedentary Behavior

**DOI:** 10.3390/ijerph19084422

**Published:** 2022-04-07

**Authors:** Tobias Engeroff, Eszter Füzeki, Lutz Vogt, Winfried Banzer

**Affiliations:** 1 Division Health and Performance, Institute of Occupational Social and Environmental Medicine, Goethe University Frankfurt, 60590 Frankfurt am Main, Germany; 2 Division of Preventive and Sports Medicine, Institute of Occupational Social and Environmental Medicine, Goethe University Frankfurt, 60590 Frankfurt am Main, Germany; fuezeki@med.uni-frankfurt.de (E.F.); banzer@med.uni-frankfurt.de (W.B.); 3 Department of Sports Medicine and Exercise Physiology, Institute of Sports Sciences, Goethe University Frankfurt, 60590 Frankfurt am Main, Germany; l.vogt@sport.uni-frankfurt.de

**Keywords:** diabetes, insulin sensitivity, hyperglycemia, depression

## Abstract

Fitness and exercise may counteract the detrimental metabolic and mood adaptations during prolonged sitting. This study distinguishes the immediate effects of a single bout vs. work-load and intensity-matched repeated exercise breaks on subjective well-being, blood glucose, and insulin response (analyzed as area under the curve) during sedentary time; and assesses the influence of fitness and caloric intake on metabolic alterations during sedentariness. Eighteen women underwent cardiopulmonary exercise testing and three 4 h sitting interventions: two exercise interventions (70% VO_2_max, 30 min, cycle ergometer: (1) cycling prior to sitting; (2) sitting interrupted by 5 × 6 min cycling), and one control condition (sitting). Participants consumed one meal with ad libitum quantity (caloric intake), but standardized macronutrient proportion. Exercise breaks (4057 ± 2079 μU/mL·min) reduced insulin values compared to a single bout of exercise (5346 ± 5000 μU/mL·min) and the control condition (6037 ± 3571 μU/mL·min) (*p* ≤ 0.05). ANCOVA revealed moderating effects of caloric intake (519 ± 211 kilocalories) (*p* ≤ 0.01), but no effects of cardiorespiratory fitness (41.3 ± 4.2 mL/kg/min). Breaks also led to lower depression, but higher arousal compared to a no exercise control (*p* ≤ 0.05). Both exercise trials led to decreased agitation (*p* ≤ 0.05). Exercise prior to sitting led to greater peace of mind during sedentary behavior (*p* ≤ 0.05). Just being fit or exercising prior to sedentary behavior are not feasible to cope with acute detrimental metabolic changes during sedentary behavior. Exercise breaks reduce the insulin response to a meal. Despite their vigorous intensity, breaks are perceived as positive stimulus. Detrimental metabolic changes during sedentary time could also be minimized by limiting caloric intake.

## 1. Introduction

Parallel to the decline in occupational-, transport-, and housework-related physical activity, a marked increase in sedentary behavior has taken place in the last decades [1]. Sedentary behavior is defined as activities with ≤1.5 metabolic equivalents of energy expenditure in a sitting or reclining posture [2]. The health consequences of prolonged sedentary behavior include an increased risk for cardiometabolic diseases [3,4] and all-cause mortality [5]. Furthermore, sedentary behavior has been suggested to negatively affect mood and well-being [6]. With the latest update in 2020, the World Health Organization emphasizes that, aside from being physically active, adults should reduce or replace time spent sedentarily as much as possible [7].

Interrupting sitting with short exercise bouts could be a further option to limit the negative effects of sedentariness in settings which do not allow replacement or reduction of sitting time [8]. Multiple meta-analyses of randomized controlled trials consistently confirmed the acute beneficial effect of exercise on markers for glucose and fat metabolism during sedentary behavior [8,9,10]. Most of the included trials analyzed the impact of sedentary behavior in men or mixed populations. Therefore, data on women is limited. In these experimental designs, the effect of exercise is analyzed using different approaches. Some compared a control condition with a trial with exercise implemented either as a single bout (usually prior to exercise) or as multiple breaks during a matched bout of sedentary time. Others analyzed the effect of physical activity by replacing a certain amount of sedentary time with activity. Findings on the beneficial influence of light-to-moderate-intensity exercise applied as breaks or replacement of sedentary time, compared to a no-exercise control, seem clear [8,9,10]. In contrast, evidence concerning the non-inferiority of a single bout of exercise compared with multiple exercise breaks is equivocal. Loh and colleagues report no significant advantages for exercise breaks over continuous exercise in their meta-analysis for light- and moderate-intensity exercise [8]. In contrast to these findings, a network meta-analysis controlled the impact of exercise intensity, and reported superior effects of exercise breaks with moderate intensity over continuous exercise with comparable intensity, and over breaks with light intensity [11]. The contradictory findings concerning the superiority of exercise breaks over continuous exercise bouts could be explained by the impact of energy expenditure. In a sub-analysis, Loh and colleagues compared only experimental designs with matched energy expenditure, and confirmed a significant advantage of exercise breaks [8]. These findings highlight the need to further investigate the impact of exercise intensity, and the balance between caloric intake and energy expenditure on the metabolic adaptations during sedentary behavior.

Current evidence thus underlines the relevance of coping strategies against the metabolic effects of prolonged sedentariness. In this regard, the available data imply that higher-intensity exercise, irrespective of the mode of application (as continuous bout or multiple exercise breaks), might be more sufficient to cope detrimental metabolic consequences [8,11]. One reason might be the increase of carbohydrate utilization during more intense exercise and subsequent sedentary behavior. Following this assumption, higher-intensity exercise might be better suited to cope with glucose and insulin spikes after meal ingestion. Furthermore, studies on multiple short bouts of vigorous exercise were already able to confirm that spreading exercise bouts over a large timeframe and matched exercise in the form of interval training could lead to comparable effects on cardiorespiratory fitness [12]. To address the current shortcomings and further elucidate the potential advantage of higher-intensity exercise breaks over matched bouts of continuous exercise, future studies need to evaluate both the impact of caloric uptake and exercise-related energy expenditure. To the best of our knowledge, to date, no study applied a design with an individualized caloric content, and therefore, was able to examine the impact of the amount of food or beverages consumed on metabolic adaptations during sedentary behavior.

Current meta-analyses discuss if anthropometrical factors, such as a low BMI, or other health indicators, such as habitual activity and physical fitness, help to prevent the detrimental effect of sedentary behavior [8]. In line with these assumptions, preliminary data already indicates that the detrimental associations between sedentary behavior and markers of cardiometabolic risk are distinctly weaker when adjusting for cardiorespiratory fitness [13]. However, so far, no experimental study included sufficient cardiopulmonary exercise testing and analyzed the impact of maximal oxygen consumption capacity on the detrimental effect of sedentary behavior, and the beneficial influence of exercise. Based on differences in muscle fiber composition, enzyme activity, and also cardiovascular adaptability, fitness could have a strong influence on the metabolic alterations induced by exercise breaks or continuous exercise [14]. Consequently, future studies need to further analyze fitness-related interindividual differences in the metabolic response. It is furthermore of utmost importance to analyze the effect of continuous and interrupted sedentary behavior on mood and well-being. Although evidence indicates that, especially, higher-intensity exercise might be feasible to increase mood and well-being in sedentary populations [15], aerobic exercise within this intensity spectrum is often criticized as unpleasant, and therefore, discussed to be not applicable within a realistic occupational setting. However, no experimental trial on metabolic effects so far asked their participants about their mood and well-being using a standardized and validated tool.

Based on the contradictory results concerning the non-inferiority of a single bout of exercise, the specific objective of this study was to assess if exercise prior to sitting leads to comparable beneficial effects on well-being, glucose, and insulin metabolism than repeated exercise breaks during sedentary behavior. Thus, our study (1) compares the effects of 4 h uninterrupted sitting with sitting interrupted with short exercise breaks (5 × 6 min) and a single energy-matched exercise bout prior to sitting (30 min) on blood glucose and insulin, as well as on well-being, and (2) analyzes the moderating effects of dietary caloric intake and cardiorespiratory fitness on metabolic alterations during sedentary behavior.

## 2. Materials and Methods

### 2.1. Trial Design

This is a randomized cross-over study with three different interventions with balanced sequence application. Participants underwent a preliminary medical examination, followed by three main interventions. This study is one part of a project evaluating the effects of sedentary behavior on glucose and fat metabolism. Findings on cholesterol and triglyceride values are published elsewhere [16]. The design, enrollment, and reporting of this study were in accordance with the Consolidated Standards of Reporting Trials (CONSORT). The study protocol was approved by the local ethic commission (121/13), and the study was registered (12 August 2021) at the German Clinical Trials Register (DRKS00010913), and was in accordance with the Declaration of Helsinki. The full trial protocol can be assessed at the German Clinical Trials Register (www.drks.de, accessed on 1 October 2021). Assessments took place at the Department of Sports Medicine at the Goethe University, Frankfurt, Germany.

### 2.2. Participants

Participants were recruited through advertisement at the university campus. All participants were female. Inclusion criteria were a BMI (kg/m^2^) between 17 and 29, and being physically active by self-declaration. General exclusion criteria were pregnancy, acute or chronic physical and psychological diseases, and drug abuse, as well as elevated fasting glucose. Sample size was calculated prior to study enrollment based on an earlier RCT [17], and revealed a total sample size of *n* = 15. Assuming a dropout rate of 20%, a minimum of 18 participants needed to be included in the study. Allocation sequence was generated using Excel for Windows. We applied a block randomization using block sizes of 6 participants. Participants did not receive information concerning their intervention sequence. Participants were asked to refrain from alcohol, caffeine, and strenuous physical activities 24 h prior to the preliminary medical examination and the three interventions, respectively. Participants were requested to maintain their weight, regular diet, and habitual physical activities for the whole duration of the study. Furthermore, participants were asked to maintain their medical contraception (if already applied), or not to start hormonal medication during study enrollment. Participants signed informed consent prior to study enrollment.

### 2.3. Preliminary Examination

A physician assessed medical history and performed an anamnesis and physical examination (including blood pressure, and heart and lung function assessments) of all participants to confirm their health status. Body weight was measured to the nearest 0.1 kg using a digital scale; height was measured to the nearest 0.1 cm using a stadiometer. Maximal exercise capacity was determined by cardiopulmonary exercise testing (CPET) using a ramp exercise test until volitional exhaustion on an electrically braked cycle ergometer. Oxygen uptake (VO_2_) was measured using a breath-by-breath gas analyzer (Oxycon Mobile, Viasys Healthcare GmbH, Hochberg, Germany). The measuring instrument was calibrated before each test using reference gases (outside air and 5% CO_2_, 16% O_2_) and automated standard ventilatory volumes (0.2 and 2 L/min). The oxygen data were reduced to 5 s stationary averages. The highest 30 s floating mean of VO_2_ within the testing time was defined as maximal oxygen uptake (VO_2_max). Individual VO_2_max data was rated as age- and sex-specific percentile values of maximal aerobic power (MAP) according to the ACSM guidelines for exercise testing and prescription [18], and provided the basis for the subsequent categorization (very poor, poor, fair, good, excellent, superior).

### 2.4. Interventions

Participants arrived in the fasted state at our facilities, and remained in this state until enrollment of the intervention. For blood sampling, a catheter was inserted into an antecubital vein. Baseline blood sampling took place after a 15 min resting period, and in the fasted state. After baseline measurement participants consumed a standardized test meal, composed according to the results of The German National Nutrition Survey [19], and consisting of white toast bread, cheese, and jam. The meal had a caloric value of 1124 kJ/100 g (268.41 kcal/100 g), and a macronutrient proportion of 51% carbohydrate, 35% fat, and 14% protein. Participants were free to choose the portion size of their meal during their first intervention. The portion size was kept constant for the other two interventions. The meal ingestion during each of the three trials took part immediately before the 4-h of sitting, and was the first bolus received after an overnight fast. Each subject completed three interventions: 1. A single bout of 30 min exercise (70% VO_2_max cycling on an ergometer) prior to uninterrupted sitting; 2. Sitting interrupted by 5 exercise breaks of 6 min ergometer cycling each (70% VO_2_max); 3. Uninterrupted sitting without exercise. The interventions were separated by a minimum of 7 days and a maximum of 21 days, and were performed in a balanced (block-randomization of sequence) design. The total sitting time was 4 h in all three trials. Sitting mimicked basic aspects of office work. Participants read, worked, or used the internet on their laptops.

### 2.5. Outcome Measures

Blood glucose and plasma insulin values were the main outcomes of this study. In each trial, insulin and glucose serum readings were taken 7 times: at baseline, at 30-, 60-, 90-, 120-, 180-, and 240-min sitting time. Venous blood samples (2 × 7.5 mL + 1 × 2.7 mL) were collected into appropriate tubes. An initial aliquot of 5 mL was discarded. For insulin measurement, samples were centrifuged at 3913× *g* for 8 min, and serum was removed and refrigerated at −20 °C subsequently. Samples were stored for later analysis at −80 °C. Enzymatic, colorimetric assays (Roche/Hitachi cobas c systems) were used to measure glucose values. Plasma insulin concentrations were obtained using direct chemiluminescent technology of the ADVIA Centaur Insulin assay (Siemens Version 128324). Glucose and insulin analyses were conducted at the Bioscientia laboratory in Ingelheim, Germany.

Subjective well-being and mood were analyzed as secondary outcomes. A validated questionnaire (mood survey) assessed subjective well-being in eight different categories [20,21]. Four of these categories were defined as positive: arousal, elevated mood, thoughtfulness, and peace of mind. The other four categories were defined as negative: anger, agitation, depression, and lack of energy. Each category was based on 5 items (40 items overall) which had to be rated using a five-point Likert scale. Participants were asked to rate their current state of well-being, and the assessments took place at the end of each intervention.

### 2.6. Statistical Methods

Statistical analysis was carried out using IBM SPSS statistics software (Version 21.0) for Windows and BIAS statistics software (Version 10.05). Descriptive statistics are reported as mean and standard deviation (SD). Homeostasis Model Assessment (HOMA) for insulin resistance (IR) index was calculated based on baseline glucose and insulin measures. Incremental area under the curve (iAUC; μU·mL^−1^·min for insulin; mg·dL^−1^·min for glucose) was calculated and time-normalized for serial data analysis of glucose and insulin using the trapezoidal method [22]. Maximal and minimal changes to baseline (μU·mL^−1^ for insulin; mg·dL^−1^ for glucose) within each trial were determined for peak data analysis. Repeated measures analysis of variance (ANOVA) or covariance (ANCOVA) and post hoc tests were used to examine differences of well-being, baseline concentrations, and differences of peak and iAUC values of glucose and insulin between interventions. The potential moderating impact of caloric intake in kilocalories and cardiorespiratory fitness, assessed as maximal oxygen uptake, on glucose and insulin measures was analyzed using Spearman correlation analyses. In case of significance, outcomes were applied as covariates for ANCOVA. The significance level was set at 5% for all tests.

## 3. Results

Out of 20 included participants, 18 completed the study. Two participants were not able to complete all three interventions due to problems with their time schedule, and thus, did not receive the allocated interventions. All included participants completed CPET until volitional exhaustion without stopping for breaks. No adverse events or medical conditions led to premature test termination of CPET or main trials. Baseline demographic and clinical characteristics, including results of CPET including calculations for energy expenditure, carbohydrate, and fat oxidation, are presented in Table 1. Maximal aerobic power (MAP) rating, according to the ACSM guidelines for exercise testing and prescription, ranged from poor to superior (median: good) (2× superior, 4× excellent; 6× good; 4× fair; 1× poor; 0× very poor).

### 3.1. Insulin and Glucose

**Figure 1:**
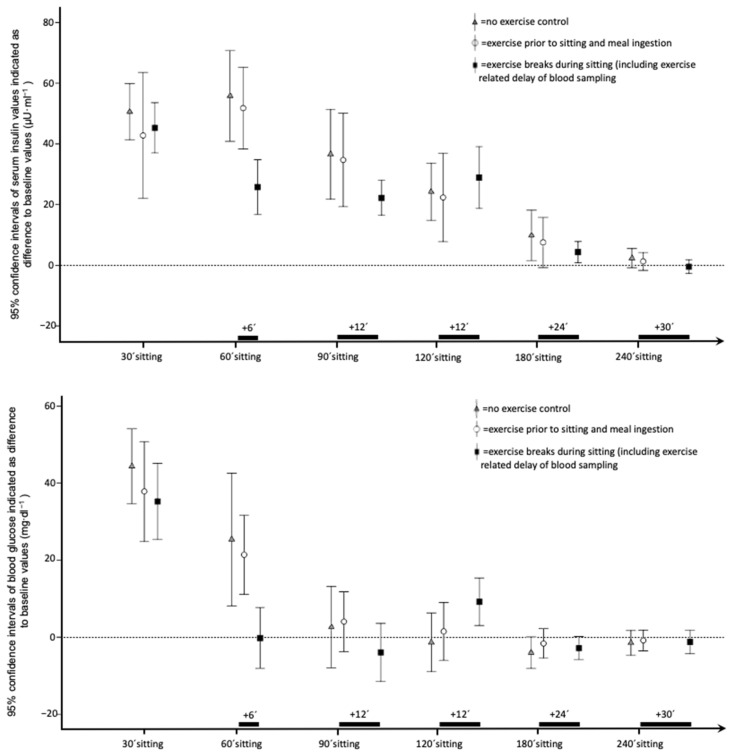
95% confidence intervals of insulin and glucose blood values of all time points during interventions with 4 h of sitting and (1) no exercise, (2) 30 min exercise prior to sitting, or (3) 5 exercise breaks with matched overall duration (5 × 6 min). Insulin values are indicated as difference to individual baseline values in microunits pro milliliter (μU·mL^−1^). Glucose values are indicated as difference to individual baseline values in milligrams per deciliter (mg·dL^−1^). Timeframe in minutes starts with meal ingestion, and is indicated on the *x*-axis (’ = minutes).

Descriptive data and detailed results of time series analysis for serum, insulin, and glucose are reported in Table 2. Figure 1 shows 95% confidence intervals of insulin values for all six time points. Repeated measures ANCOVA of maximal insulin differences to baseline and incremental area under the curve indicated significant between-intervention effects. Furthermore, ANCOVA revealed a significant moderating influence of caloric intake on insulin values during sitting, and on the intervention effects. Post hoc tests indicated a significant effect of exercise breaks compared to both the control condition and the exercise prior to sitting trial. The effect of exercise prior to sitting did not reach statistical significance for both insulin outcomes. Figure 1 shows 95% confidence intervals of glucose values for all time points. As indicated in Table 2, no between-intervention effects for glucose data were detected. A significant between-participants effect of caloric intake, and thus, an influence of the amount of caloric intake on elevated glucose levels during 4 h of sitting, was confirmed. ANCOVA showed no effect of intervention on minimal differences to baseline for glucose and insulin.

### 3.2. Cardiorespiratory Fitness and Caloric Intake

Dietary caloric intake was 2176 kJ ± 886, and in relation to bodyweight, 37 kJ/kg ± 15 (519.7 kcal ± 211.5; 8.9 kcal/kg ± 3.7). Participants ingested 66.06 g ± 26.89 glucose, 20.5 ± 8.34 fat, and 18.22 g ± 7.42 protein. The balance between ad libitum intake and exercise-related energy expenditure indicated that 58.4% ± 27.1 of the meal caloric content was burned during exercise. Based on CPET data, at 70% VO_2_max, the predominant energy source during physical activity was carbohydrate. Consequently, mean carbohydrate uptake during the ad libitum meal and expenditure during exercise was close to balance (+3.3 g ± 27.5). Table 2 indicates insulin and glucose baseline values and time series data, including maximal and minimal changes to baseline and incremental area under the curve, for each intervention. Baseline values showed no difference in fasting concentrations of insulin or glucose between trials. A priori Spearman correlation analysis indicated an association of dietary caloric intake with incremental area under the curve of insulin (exercise breaks: r = 0.787; exercise prior: r = 0.823; control: r = 0.822; *p* < 0.01) and glucose (exercise breaks: r = 0.501; *p* = 0.034; exercise prior: r = 0.484; *p* = 0.042; control: r = 0.533; *p* = 0.023) of all interventions. Cardiorespiratory fitness was not associated with glucose or insulin data.

### 3.3. Mood and Well-Being

Descriptive data and detailed results of ANOVA for exercise effects on well-being and mood are reported in Table 2. Both exercise interventions did not lead to detrimental changes in any of the positive or negative categories. Exercise breaks induced beneficial effects on arousal, and led to lower levels of agitation and depression during sedentary behavior compared to a no-exercise control. Exercise prior to sitting led to a comparable decrease in agitation than exercise breaks, and to an increase in peace of mind.

## 4. Discussion

The insulin response to an ad libitum meal during 4 h of sedentary behavior is lower when sitting is interrupted with short exercise breaks (5 × 6 min). Furthermore, our data indicates that these metabolic effects are accompanied by a positive subjective response. Exercise prior to sitting was not feasible to induce comparable beneficial metabolic effects. Additionally, we analyzed the impact of cardiorespiratory fitness and the caloric content of an ad libitum meal during sedentary behavior. This experiment underlines the influence of caloric intake not only on glucose and insulin spikes during postprandial sedentariness, but also on the insulin-lowering effect of exercise breaks. Our sample showed a broad range of cardiorespiratory fitness. However, fitness had neither an impact on detrimental changes in glucose or insulin metabolism during sedentary behavior, nor on the beneficial effect of exercise breaks.

In line with the assumption made in a recent network analysis [11], we can confirm a superior effect of exercise breaks compared to an energy-expenditure- and intensity-matched single bout of exercise on insulin metabolism during sedentary behavior. In our study, five exercise breaks of six minutes cycling with vigorous intensity during four hours of sitting led to 30% lower insulin iAUC values compared to a control condition with no exercise. The glucose response to an ad libitum meal before sitting was not significantly altered by exercise in any form. This might be due to large interindividual differences in glucose response, and underlines the relevance of caloric intake for both the detrimental influence of sedentary behavior, and the beneficial impact of exercise. Contrary to meta-analytic evidence on the metabolic response to predefined caloric boli [8,9,10], we cannot confirm a significant lowering effect on postprandial glucose levels for settings in which individuals can choose the amount of food they ingest during sedentary behavior. Since most of the earlier RCTs included mixed populations or men, it needs to be kept in mind that these differences could be influenced by our female study sample. In line with earlier findings, our data support a potential larger effect of higher-intensity exercise [11]. Furthermore, we confirm the impact of caloric balance on the beneficial impact of interruptions during prolonged sitting which was previously discussed by Loh and colleagues [8].

We assessed two influencing factors in our study. Previous experiments already examined the influence of variations in caloric intake on insulin action during prolonged sitting [23]. Our experiment extends these findings concerning the impact of caloric intake on the beneficial effect of exercise breaks during sedentary behavior. Our data thus show that both the insulin response to food intake during sitting and the insulin-lowering effect of exercise breaks are related to the number of calories ingested. This could partially explain why ad libitum food intake did not lead to a more hyperglycemic status during prolonged sitting in our experiment, and thus, why breaks induced changes solely in insulin release. Consequently, adjusting the timing or amount of food and beverage intake during sedentary behavior could be an important intervention strategy to mitigate detrimental metabolic effects.

Our participants’ cardiorespiratory fitness level ranged from poor to excellent according to ACSM’s percentile values [18]. Observational data indicate that the detrimental associations between sedentary behavior and markers of cardiometabolic risk are distinctly weaker when adjusting for cardiorespiratory fitness [13]. Furthermore, a recent meta-analysis discusses that the beneficial exercise effects on postprandial metabolism during sitting might be related to fitness or body composition [8]. In our sample of female humans (with a close age and BMI range), fitness did not influence acute metabolic changes during prolonged sitting in postprandial state. Consequently, future studies need to analyze whether body composition or other modifiable factors which are related to fitness influence metabolic reactions during sedentary time.

The exact mechanisms behind the effects of breaking up sedentary behavior remain to be unraveled. Based on bed rest and animal studies, Hamilton and colleagues were one of the first to suggest that a decrease in metabolic processes during rest might lead to lower insulin-independent clearance of glucose from the bloodstream [14]. Mechanisms responsible for the lower insulin response include decreased insulin-independent GLUT-4 glucose transporter expression on the cell membrane, stimulated by the lack of muscle contraction, as well as decreased enzyme-linked substrate metabolization and skeletal muscle blood flow [14]. In line with others, we observed that continuous exercise and breaks during sedentary behavior had different acute effects on insulin concentration [11]. Our findings support the hypothesis that breaks, but not a single exercise bout, can maintain enzymatic function or attenuate degradation of insulin-independent glucose transporters during prolonged sitting. Based on our results, it is not possible to determine whether increased intracellular turnover or glucose storage enables insulin-independent glucose uptake of muscle cells. Additionally, exercise-induced mechanisms, such as excess oxygen consumption (EPOC), replenishment of the phosphagen system (adenosine triphosphate, creatine phosphate), and lactate processing [24] occur multiple times during sitting interrupted by multiple vigorous exercise bouts. Yet, bearing in mind the magnitude of such post-exercise adaptations, it is unlikely that these mechanisms have a clinically relevant impact [24,25]. We therefore hypothesize that metabolic effects observed in our and other studies may indicate an exercise-induced shift in substrate utilization from fat and glucose as the main sources to predominantly glucose during both breaks and subsequent sitting.

Our study is the first randomized controlled study which analyzed the effect of vigorous-intensity exercise interventions on mood and well-being during sedentary behavior. Neither a single exercise bout prior to sitting nor exercise breaks during sitting led to lower ratings of well-being during sedentary behavior compared to a no-exercise control. Both intervention forms led to lower self-perceived agitation. Continuous exercise in the morning also induced a greater feeling of peace of mind. Breaking sedentariness with vigorous-intensity exercise bouts induced higher arousal and lower levels of depression after 4 h of sitting. Against anecdotal evidence, these findings indicate that both interventions are perceived as a positive experience when applied in a sedentary setting. In line with an early review on experimental studies, our data confirm the positive effects of a single bout of exercise with moderate-to-vigorous intensity on mood and well-being [26]. An observational study on break patterns during sedentary behavior reports an association of break intensity and frequency with calmness and arousal during sedentary behavior [27]. Likewise, first experimental studies revealed that breaks during sedentariness were associated with higher self-perceived rigor and lower levels of fatigue [28,29]. Our experiment is the first to confirm a causal link between breaks and beneficial changes in mood and well-being using a standardized and validated tool for the assessment of positive and negative dimensions of mood [20,21]. In line with an earlier experiment which applied moderate-intensity exercise, we were able to detect specific effects of exercise breaks compared to a matched single bout of exercise. Although Bergouignan and colleagues [28] did not detect significant effects of moderate-intensity exercise breaks on cognitive function in this earlier experiment, one might speculate that increases in arousal after short exercise bouts with higher intensity might also lead to beneficial alterations of cognitive performance [30]. Future studies need to further evaluate the effect of exercise breaks on mood and cognitive performance in real-life settings such as office work.

A strength of our study is its rigorous, counter-balanced intervention order, and the use of an ecologically valid approach with a self-selected amount and realistic macronutrient composition of female-specific daily intake in the form of a typical western breakfast [19]. Furthermore, we applied a standardized sitting time of 4 h during all trials instead of replacing sitting time with exercise. This approach allowed us to compare the metabolic influence of a comparable amount of sedentary behavior with and without exercise. Further, we used gas-analysis-based cardiorespiratory performance testing, which is the gold standard to establish exercise intensity, and assess VO_2_max. This method allowed us a higher level of standardization of interventions than in previous studies. The inclusion of premenopausal healthy females allowed us to examine a homogeneous study population. Although participants were allowed to continue hormonal contraception, we asked to maintain the dosage and method of application during study enrollment. Hormonal influences are mitigated by our randomized and balanced design with a limited timeframe on a maximal wash-out period of 3 weeks between trials. A limitation of our study is that we did not assess body composition, and habitual dietary and physical activity levels using validated instruments. These factors might have an influence on metabolic adaptations to sedentary behavior and acute exercise effects. In a sample with BMI values ranging around 21 ± 2, the measurement of cardiorespiratory fitness, however, might be even more relevant. We applied vigorous-intensity exercise in order to generate a sufficient stimulus for glucose metabolism and overall energy turnover within a minimal period of time, and to answer the question of whether vigorous-intensity exercise prior to sitting might be non-inferior to multiple exercise bouts, and thus, sufficient to counteract unfavorable metabolic changes during prolonged sitting.

## 5. Conclusions

In summary, we showed that in a realistic setting with a meal mimicking a typical western breakfast and a sedentary period of 4 h (simulating half a workday until lunch break), regular short exercise breaks, but not exercise prior to sitting, can lower blood insulin levels in premenopausal, healthy, female participants. Caloric intake strongly influences metabolic regulation, and must be considered when interpreting our and other findings. In our sample, cardiorespiratory fitness had no influence on changes of cardiometabolic markers. Direct comparison of participants with high vs. low cardiorespiratory fitness in larger study samples might yield more insight whether, and if yes, to what extent, fitness can protect from the acute negative health effects of sedentary behavior. Our results confirm that for good health and good mood, not only staying active according to recommendations, but also breaking up long periods of sitting, is of high relevance. Physically active breaks, even with vigorous intensity, are perceived as a positive influence during prolonged sitting. We suggest that public health recommendations include advice to interrupt prolonged sitting if a reduction or replacement cannot be realized. Our study also suggests that limiting food and beverage intake with high carbohydrate content during sedentary behavior might mitigate negative metabolic effects.

## Figures and Tables

**Table 1:** Baseline demographic and clinical characteristics, including results of the cardiopulmonary exercise testing including measures of energy expenditure.

Anthropometrics	*n* = 18	Mean Values ± Standard Deviation 95% Confidence Interval
Age (Years)	25.6 ± 2.6 24.3–26.9	
Height (Meter, m)	1.66 ± 0.07 1.63–1.69	
Weight (Kilogram, kg)	59.5 ± 9.0 55.0–64.0	
Body Mass Index (BMI, kg/m^2^)	21.5 ± 2.0 20.5–22.5 (Minimum: 17.5; Maximum: 25.6)	
Fasting Glucose (Milligrams per deciliter, mg·dL^−1^)	75.8 ± 6.8 79.6–72.0	
HOMA-IR	1.0 ± 0.3 0.8–1.2	
**Cardiopulmonary Exercise Testing**	**70% VO_2_max**	**VO_2_max**
VO_2_ (Liters per minute, L/min)	1.7303 ± 0.2993 1.5815–1.8791	2.4475 ± 0.3811 2.2580–2.6370
VO_2_ (Milliliters per kilogram bodyweight per minute, mL/kg/min)	29.2 ± 3.6 27.4–31.0	41.3 ± 4.2 39.2–43.4
VCO_2_ (Liters per minute, L/min)	1.8109 ± 0.3242 1.6497–1.9722	3.1850 ± 0.4210 2.9757–3.3944
Respiratory Quotient (VCO_2_/VO_2_)	1.05 ± 0.05 1.02–1.7	1.31 ± 0.05 1.28–1.33
Heart rate (beats per minute)	161 ± 9 156–165	190 ± 9 186–194
Power (Watt)	140 ± 28 127–154	249 ± 31 233–264
Metabolic Equivalent of Task (Kilocalories per kilogram bodyweight per hour, kcal/(kg·h)	9.27 ± 1.16 8.70–9.84	13.11 ± 1.36 12.43–13.79
**Energy Expenditure for 30 min exercise at 70% VO_2_max**:		
Energy Expenditure (Kilocalories, kcal)	261.68 ± 45.40 239.10–284.26	370.58 ± 57.71 341.88–399.27
Carbohydrate Oxidation (Grams)	30.69 ± 12.66 24.40–37.00	62.80 ± 11.82 56.93–68.68
Fat Oxidation (Grams)	5.77 ± 4.58 3.50–8.05	0.44 ± 1.32 0.21–1.10

**Table 2:** Left columns: descriptive data for blood insulin in microunits per milliliter (μU/mL) and glucose in milligrams per deciliter (mg/dL), indicated as baseline, maximal and minimal difference to baseline, and descriptive data for mood and well-being. Incremental area under the curve (iAUC) for insulin, indicated as microunits per milliliter per time in minutes (μU/mL·min) and, for glucose, indicated as milligrams per deciliter per time in minutes (mg/dL·min). For mood and well-being, all positive (arousal, elevated mood, thoughtfulness, peace of mind) and negative outcomes (anger, agitation, depression, lack of energy) are indicated. Mean value and standard deviation are given for all outcomes in all trial conditions (exercise breaks, prior exercise, and no exercise control). Right column: results of repeated measures ANCOVA for within-subject (intervention), between-subject (caloric intake), and interaction effects. Level of significance is *p* = 0.05. Significant results er indicated by bold font * = significant difference to no exercise control, # = significant difference to trial with prior exercise.

	Trial Condition (4 h Sitting with)			Two Factorial Repeated Measures ANCOVA A: Within Subject Effect of Trial Condition B: Interaction Effect C: Between Participants Effect of Caloric Intake
	Exercise Breaks	Exercise Prior to Sitting	No Exercise	
**Insulin**				
Baseline	6.8 ± 2.7	6.5 ± 2.3	6.3 ± 2.4	A: *p* = 0.414; F = 0.91; df = 1.87 B: *p* = 0.265; F = 1.39; df = 1.87 C: *p* = 0.844; F = 0.40; df = 1.00
Maximal difference to baseline	46.8 ± 14.9 *#	61.9 ± 37.7	62.9 ± 27.1	**A: *p* = 0.012; F = 5.36; df = 1.85** **B: *p* = 0.001; F= 8.60; df = 1.85** **C: *p*** < **0.000; F = 26.09; df = 1.00**
Minimal difference to baseline	−0.2 ± 4.6	1.2 ± 5.58	1.8 ± 6.0	A: *p* = 0.728; F= 0.32; df = 2.00 B: *p* = 0.104; F= 2.65; df = 2.00 **C: *p*** < **0.000; F = 43.96; df = 1.00**
Incremental area under the curve	4057.2 ± 2079.8 *#	5346.7 ± 5000.7	6037.0 ± 3571.0	**A: *p* = 0.003; F= 7.23; df = 1.93** **B: *p*** < **0.000; F= 11.14; df = 1.93** **C: *p*** < **0.000; F= 75.70; df = 1.00**
**Glucose**				
Baseline	76.7 ± 6.0	76.1 ± 6.5	76.6 ± 6.6	A: *p* = 0.747; F = 0.24; df = 1.68 B: *p* = 0.815; F = 0.16; df = 1.68 C: *p* = 0.619; F = 0.26; df = 1.00
Maximal difference to baseline	35.4 ± 16.2	42.3 ± 23.0	47.0 ± 22.2	A: *p* = 0.282; F = 1.32; df = 1.94 B: *p* = 0.080; F = 2.76; df = 1.94 **C: *p* < 0.000; F = 23.08; df = 1.00**
Minimal difference to baseline	−11.6 ± 10.8	−9.2 ± 7.3	−11.3 ± 10.1	A: *p* = 0.279; F = 1.33: df = 1.84 B: *p* = 0.380; F = 0.98; df = 1.84 C: *p* = 0.092; F = 3.21; df = 1.00
Incremental area under the curve	957.9 ± 1735.0	2144.0 ± 2399.4	1828.3 ± 2956.9	A: *p* = 0.894; F = 0.71; df = 1.57 B: *p* = 0.475; F = 0.70; df = 1.57 **C: *p* < 0.000; F = 25.27; df = 1.00**
**Mood and Well-Being**				
**Positive Outcomes**				**Repeated measures ANCOVA** **Within subject effect of intervention (df = 2)**
Arousal	12.4 ± 3.2 *	11.6 ± 4.7	9.9 ± 3.3	***p* = 0.05; F = 3.27**
Elevated Mood	15.8 ± 3.5	15.2 ± 4.4	13.2 ± 4.2	*p* = 0.058; F = 3.10
Thoughtfulness	8.3 ± 2.3	8.6 ± 2.8	8.9 ± 3.8	*p* = 0.637; F = 0.46
Peace of Mind	14.7 ± 3.5	15.2 ± 3.4 *	12.8 ± 4.4	***p* = 0.022; F = 4.25**
Negative Outcomes:				
Anger	6.4 ± 1.8	6.1 ± 1.8	7.4 ± 2.8	*p* = 0.105; F = 2.41
Agitation	8.6 ± 3.3 *	8.7 ± 2.5 *	11.4 ± 4.9	***p* = 0.003; F = 6.74**
Depression	6.4 ± 1.8 *	7.4 ± 3.0	8.7 ± 3.6	***p* = 0.033; F = 3.76**
Lack of Energy	11.9 ± 4.0	14.3 ± 5.4	15.2 ± 6.1	*p* = 0.056; F = 3.14

## Data Availability

The data set for meta-analytic calculations will be made available via direct contact with the investigator, and after approval of a written proposal and a signed data access agreement.
